# Risk Factors and Spatiotemporal Distribution of Lumpy Skin Disease Occurrence in the Asian Continent during 2012–2022: An Ecological Niche Model

**DOI:** 10.1155/2023/6207149

**Published:** 2023-05-11

**Authors:** Yuepeng Li, Qi An, Zhuo Sun, Xiang Gao, Hongbin Wang

**Affiliations:** ^1^College of Veterinary Medicine, Northeast Agricultural University, Harbin, China; ^2^Key Laboratory of the Provincial Education Department of Heilongjiang for Common Animal Disease Prevention and Treatment, College of Veterinary Medicine, Northeast Agricultural University, Harbin, China

## Abstract

Lumpy skin disease (LSD) is an emerging transboundary infectious disease of animals with high morbidity and low mortality rates. The infection occurs in cattle, buffalo, and some closely related wild animals, with cattle and buffalo showing higher susceptibility than other species. The primary mode of disease transmission is the mechanical dispersion of bloodsucking insects. The disease symptoms, including animal fur damage, weight loss, decline in milk production, infertility, and miscarriage, lead to huge economic losses in regions and countries with LSD outbreaks. The present study aimed to analyze the incidence data of LSD in the Asian continent from January 2012 to September 2022, identify spatiotemporal clusters and risk factors of the disease, and establish a maximum entropy ecological niche model to predict high-risk areas for disease outbreaks. The studied variables included bioclimatic factors, land type, and population density. Following the screening process, 12 variables were included in the maximum entropy model. Among them, the variable contribution rates of cattle density, land cover, isothermality, buffalo density, and maximum temperature of the warmest month were 53.8%, 10.9%, 9.2%, 8.9%, and 8%, respectively. Accounting for more than 90% of the total variable contribution rate, these five variables were considered to be the important influencing factors of LSD outbreaks. According to the results, nine spatiotemporal clusters approximately matched the high-risk areas predicted by the model. The Caucasus region of Russia; the Russian border areas of Kazakhstan, Turkey, Syria, Lebanon, Palestine, and Israel; and the western regions of Iran, India, and Southeast Asia were predicted to be high-risk areas. Thus, this study provides the spatiotemporal clusters, risk factors, and high-risk areas of LSD outbreaks in the Asian continent, which can help formulate more effective disease prevention and control policies.

## 1. Introduction

Recently, large outbreaks of lumpy skin disease (LSD) have been reported in previously disease-free areas such as Thailand [1], Nepal [2], Vietnam [3], and India [4]. These regions have witnessed a surge in the number of cases of this disease, and a new transboundary epidemic is emerging [5].

LSD is a transboundary infectious animal disease caused by the LSD virus. This highly species-specific virus infects only cattle, buffalo, and other closely related wildlife, with cattle being more susceptible to this disease than other species. The disease incidence is very high and can reach 100%, but the mortality rate is low, usually below 5%, and can reach 20% in exceptional situations. Although the disease affects animals of all age groups and both genders, younger animals show greater susceptibility to the disease [6].

The primary clinical signs of LSD are high fever (biphasic fever); increased oral, nasal, and ocular discharge; declined milk production; depression; abortion; and infertility. The infected animal develops multiple, large, and hard skin nodules throughout the body, which may rupture and secrete pus, leading to skin damage and increased risk of transmission; moreover, necrotic lesions of the respiratory and digestive tracts can lead to prolonged anorexia, resulting in chronic wasting of the animal. The disease can cause damage to the immune system, resulting in mastitis and uterine inflammation and other complications, leading to reproductive disorders in dairy cows [7, 8]. Skin damage, weight loss, reduced milk production, infertility, and abortion are the critical outcomes of the disease, leading to huge economic losses [9, 10].

LSD transmission shows a significant seasonal pattern [11]. The mechanical spread of arthropods explains well the phenomenon of LSD transmission. Airborne arthropods are an important vector of transmission of LSD [12], and most bloodsucking insects can fly up to 100 m in the absence of air current movement [13]; however, because virus is often carried in the insect's mouthparts, the direction and force of the wind may cause the LSD virus to spread over long distances following the insects' movement [14, 15]. Although this transmission process has not yet been confirmed, many studies have shown that a variety of blood-sucking arthropods can act as mechanical vectors of disease transmission, including *Stomoxys calcitrans* and *Aedes aegypti* [16, 17]. Tuppurainen et al. [18] found that the tick *Rhipicephalus appendiculatus* that transmits the virus by feeding on the damaged skin of infected animals can mechanically transmit the LSD virus [19]. This is the first evidence of transmission of the LSD virus by ticks. The widely distributed stable fly (*Stomoxys* species) is thought to be the primary vector of LSD virus transmission. Issimov et al. [20] demonstrated that three *Stomoxys spp.* (*Stomoxys calcitrans*, *Stomoxy sitiens*, and *Stomoxys indica*) can transmit the LSD virus. Nonbiting flies also have great potential for transmission, and latent LSD virus has been detected in these flies [21]. Trade and mass migration facilitate the spread of the disease; for example, the influx of refugees from war zones, trafficking of livestock, and long-distance migration of cattle are considered crucial factors for the increased prevalence of LSD in some areas [22]. The delay in reporting LSD cases due to the lack of awareness of the disease among farmers and the migration to a nomadic or seminomadic lifestyle have largely increased the difficulties in combating this disease [23].

An outbreak of LSD was first reported in Zambia in 1929, and the disease subsequently became endemic throughout the African continent. In 2006, a widespread outbreak of LSD occurred in the Middle East. This was the first instance of LSD reported outside the African continent, and as the epidemic traveled further, it spread to South East Europe, the Balkans, and the Russian Caucasus [24]. The disease was first reported in the Iran and the Russian Federation in 2014 and 2015, respectively [25]. LSD cases were first reported in China and India in 2019 [4, 26]. The disease was first reported in Thailand in 2020; the virus isolated from the diseased animals had the same genotype as that reported in China (2019) and showed a similar homology to the virus isolated from Russia (2017) [1]. Subsequently, widespread outbreaks were reported in various countries in Southeast Asia and India.

The current LSD epidemic poses a serious threat to the Asian continent. There are three main reasons for this threat: (1) the current mode of disease transmission between animals and the variations in its transmission pattern under different ecological and climatic conditions are not fully understood, especially because the mechanical transmission process of the vector remains unknown [27]; (2) LSD is a new transboundary infectious disease, and most veterinary staff and farmers do not have sufficient knowledge of this disease, resulting in several cases of underreporting and misreporting, all of which hinder with effective prevention strategies; and (3) a lack of efficient and reasonable vaccines for LSD. The only commercially available live attenuated virus vaccine for LSD is currently banned in many countries (such as the Russian Federation), and vaccines for goat pox and sheep pox are effective but have certain risks associated with their administration [24, 28].

Because of the delayed nature of disease epidemic reporting by the World Organization for Animal Health (OIE; https://www.oie.int/), the present study analyzed the data on LSD epidemics in Asian continent reported between January 2012 and September 2022 to characterize the spatial and temporal distribution of LSD in the Asian continent.

This study identifies the present time as the temporal scale and Asian continent as space scale, developed an existence-only maximum entropy model to analyze and determine the important risk factors influencing LSD outbreaks, and mapped high-risk areas of present time LSD outbreaks. At the same time, the spatiotemporal clustering analysis was used to review trend in LSD outbreaks across the Asian continent over the past decade. It provides a strong basis and support for the prevention and control of LSD in the Asian continent.

## 2. Materials and Methods

### 2.1. Study Region

The entire Asian continent was considered the study region for this research. Because most of Russia's land area is located within the Asian continent and is closely connected to most Asian countries, entire Russia was included in the study without considering the division of national boundaries and political factors to ensure the integrity of the study region. Although our study area also included a small part of the European country, for example, Turkey, it is part of the Asian continent in terms of geographical division and we chose to include in the study scope.

### 2.2. Data Source

LSD is listed as a global notifiable animal disease by the OIE (World Organization for Animal Health) (https://www.oie.int/). The data of the reported outbreaks included the location, region, latitude and longitude coordinates, species of infected animals, number of cases, and other relevant information. From January 2012 to September 2022, 2322 cases of LSD were reported by the OIE in the study area, and the case points were examined individually to ensure that the reported incidence locations were within the study region. To reduce spatial clustering, the rangeBuilder package in R software was used to filter out the spatial points with a filtering range of 10 km, and duplicate points were eliminated. A total of 1727 case reports were included in the final model.

### 2.3. Variable Acquisition and Processing

Different ecological and climatic conditions affect the duration of survival of LSD viruses in the nature and the speed and extent of transmission in animal populations [29]. Hence, in the present study, the bioclimatic variables, land type, normalized difference vegetation index (NDVI), and livestock density were selected by considering three aspects: climatic factors, land type, and host (Tables 1 and 2).

Climatic factors included 19 bioclimatic variables, wind speed, and solar radiation. Land type variables included the NDVI and land cover. These variables influence the survival and spread of viruses and vectors in the environment. Livestock density included cattle and buffalo density. The climatic factors were derived from the WorldClim 2.1 database and may adequately reflect the current global bioclimatic conditions. The 19 bioclimatic variables were tested for multicollinearity and Pearson's correlation coefficients using the USDM package in *R* software. Variables with correlation coefficients greater than 0.7 and variance inflation factor (VIF) greater than 5 were excluded [30], and 6 bioclimatic variables were finally retained. The NDVI was obtained from the National Tibetan Plateau Third Pole Environment Data Center (https://data.tpdc.ac.cn/en/data/9775f2b4-7370-4e5e-a537-3482c9a83d88/). The data on land cover types were obtained from the European Space Agency Climate Change Initiative project (https://maps.elie.ucl.ac.be/CCI). Livestock densities were obtained from the Gridded Livestock of the World (https://www.fao.org/land-water/land/land-governance/land-resources-planning-toolbox/category/details/en/c/1236449/) provided by the Food and Agriculture Organization of the United Nations. Finally, 12 variables were identified for inclusion in the model (Table 3).

The raster data acquired from different sources had different pixel sizes. By using ArcGIS software, the raster data were uniformly cropped to the extent of the Asian continent, and the raster variables were adjusted to a uniform cell size (5 arc minute resolution) and coordinate system by using the resampling tool.

### 2.4. Spatiotemporal Clustering Analysis

Spatiotemporal clustering analysis was performed to investigate the significant spatial aggregation regions and periods of LSD in the Asian continent. Spatiotemporal analysis was chosen as the analysis method, while spatiotemporal rearrangement was used for the probability model; moreover, this method requires only case information and does not require risk population data [31]. Within the maximum extension of the spatial window, the maximum radius was set to 1000 km, and 50% of the study period was set as the maximum time window. The final Monte Carlo random sampling method was used to calculate the *P* value, and the spatial aggregation regions with *P* < 0.01 were considered statistically significant. The number of Monte Carlo sampling iterations was 999. The spatiotemporal clustering analysis was performed in SaTScan v9.6 software.

### 2.5. Construction of an Ecological Niche Model

A maximum entropy ecological niche model was used to predict the suitable areas for LSD outbreaks and the critical risk factors affecting LSD outbreaks in Asian continent. The maximum entropy model is an existence-only ecological niche model in which 10,000 background points are randomly selected as pseudomissing data in the model. The filtered 1727 LSD outbreak locations were divided into two parts: 80% as the training set and 20% as the test set. To prevent overfitting of the model, the regularization constant was set to 1, and the model was run 10 times, with the final result obtained as the mean average value of the 10 runs. The area under receiver operating characteristic (AUROC) values was used to assess the predictive power of the model, and the significance of each variable was assessed using Jackknife plots and the percentage contribution of the variables. The final binary risk map was generated with values closer to 0 indicating that the area is not suitable for disease outbreaks and the value of 1 representing the most suitable areas for disease outbreaks. MaxEnt version 3.2.0 was used for model development and ArcGIS 10.2 software was used for final visualization.

## 3. Results

### 3.1. Spatiotemporal Clustering Analysis

**Figure 1:**
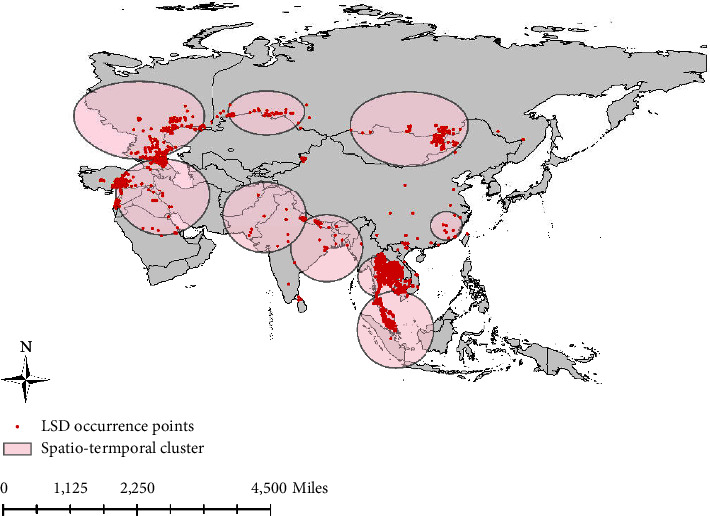
The LSD epidemic outbreak point and spatiotemporal clusters areas in the Asian continent (January 2012 to September 2022).

Nine spatiotemporal clusters were identified across the Asian continent. The first cluster included 467 cases from 2014 to 2016 in the Middle East; the second temporal cluster included 202 cases from 2016 to 2018 in the western region of Russia; and the third temporal cluster included 50 cases from 2018 to 2019 in the border area between Russia and Kazakhstan. The fourth temporal cluster included 38 cases from 2019 to 2020 in the southeast coastal region of China; the fifth temporal cluster included 25 cases from 2019 to 2020 in eastern India, Nepal, Bhutan, and Bangladesh; and the sixth temporal cluster included 108 cases from 2021 to 2022 in Mongolia. The seventh temporal cluster included 19 cases from 2021 to 2022 in northwest India and Pakistan; the eighth cluster included 439 cases from 2021 to 2022 in Southeast Asia; and the ninth cluster included 363 cases from 2021 to 2022 in Malaysia (Table 4) (Figure 1).

### 3.2. Maximum Entropy Model Evaluation and Effect of Chosen Variables

**Figure 2:**
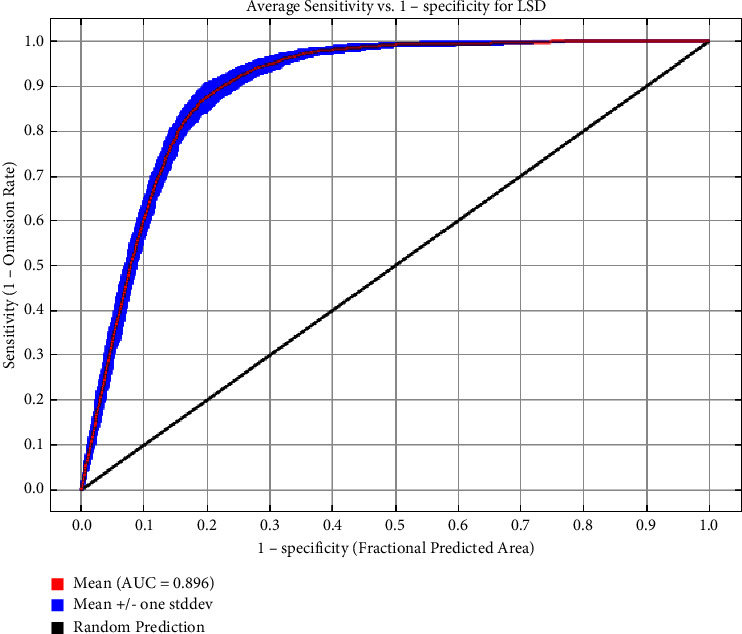
Receiver operating characteristic curve (ROAUC) plot of LSD MaxEnt model.

**Figure 3:**
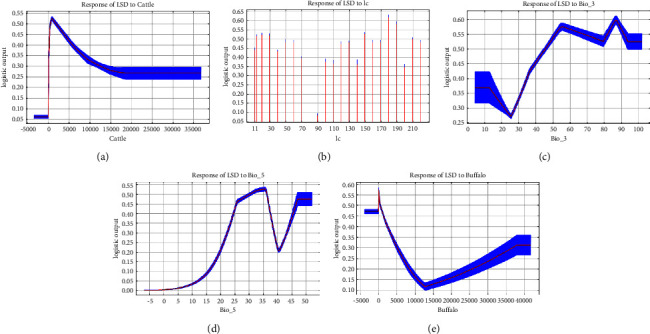
Response curve of important variables: (a) cattle (cattle density); (b) Lc (land cover); (c) Bio_3 (isothermality); (d) Bio_5 (max temperature of warmest month); and (e) buffalo (density of buffalo).

**Figure 4:**
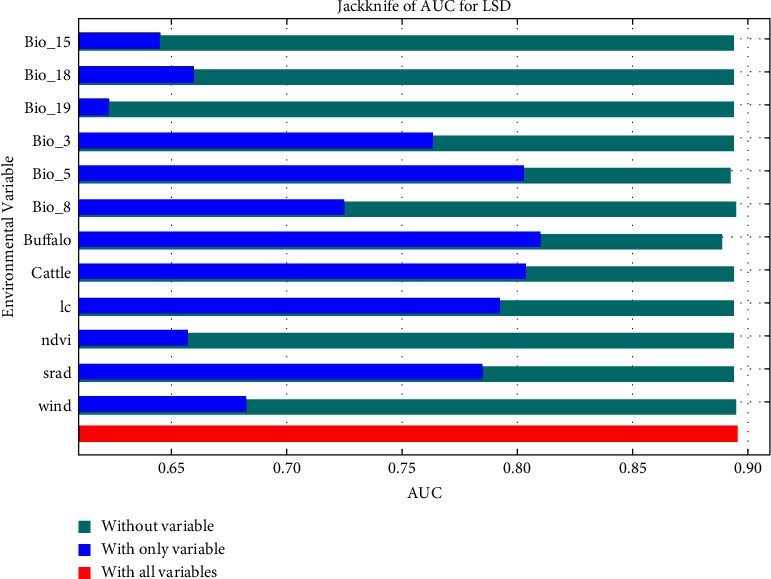
Jackknife plot of LSD MaxEnt model.

The average AUROC value after 10 runs of the model was 0.895 (Figure 2), indicating that the model performed well and could adequately differentiate between suitable and unsuitable areas of the LSD outbreaks. Next, all variables were screened for their contribution to disease occurrence, and 12 variables were finally included in the model. The variable with the highest contribution was cow density (53.8%), followed by land cover (10.9%), isothermality (9.2%), buffalo density (8.9%), and maximum temperature of the warmest month (8%); these five variables accounted for over 90% of variable contribution rates (Table 5). The variation characteristics of the important variables and the ranking of importance are smoothly represented by percentage contribution of the variables (Figure 3) and Jackknife plots (Figure 4).

**Figure 5:**
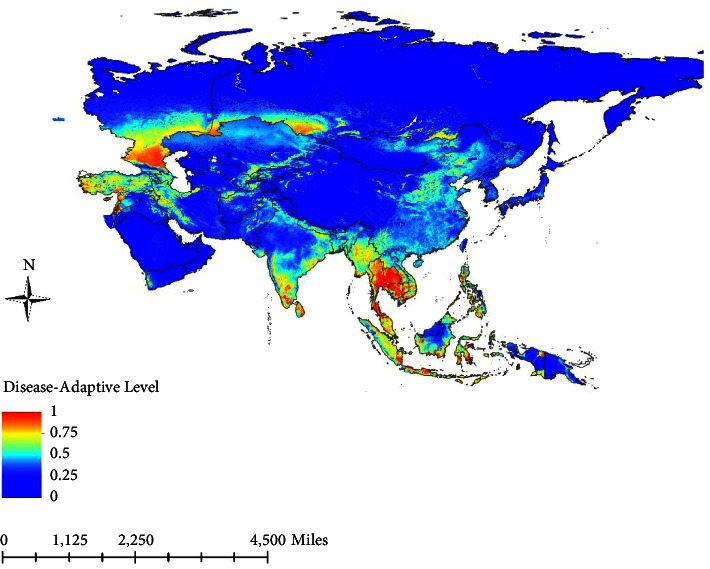
The risk level of LSD outbreaks in the Asian continent (0 represents no risk, 0.25 represents low risk, 0.5 represents medium risk, 0.75 represents high risk, and 1 represents the highest risk).

ArcGIS was used to visualize the results, and the suitable and unsuitable areas for disease outbreaks were classified as high-risk and low-risk areas, respectively. High-risk zones of LSD outbreaks on the Asian continent include the Caucasus region of Russia, areas of the Russian border with Kazakhstan, the central and western areas of Turkey, the western coastal and south-western border areas of Syria, the southern areas of Lebanon, the northern and central areas of Israel, all of Cyprus and Palestine, Iran's northern border as well as the western border areas and the northwestern border areas of Iraq, the border areas between Afghanistan and Turkmenistan, the border areas between Kazakhstan, Kyrgyzstan, and Uzbekistan, the south-western border areas of Nepal, the southern and eastern parts of India, and parts of Southeast Asia including central Myanmar, most areas of Thailand and Cambodia, the south-western border of Laos, the southern part of Vietnam, the Malay Peninsula, the Indonesian island of Sumatra, and the island of Java. A notable finding was the presence of a certain risk of disease outbreaks in northeast China and some countries in West Asia, although there have been no reports of the disease in these regions (Figure 5).

## 4. Discussion

Since the first discovery of LSD in Africa in 1921, large-scale outbreaks of LSD have occurred in Africa, Asia, and Southeast Europe [10, 32], and this disease has received considerable attention in the international community for developing effective disease prevention measures. Recently, large-scale outbreaks of LSD have been reported in Indian media; however, because of the delay of the OIE report, the outbreak data of India in September 2022 are not included in this article. In the present study, based on the incidence data of LSD from January 2012 to September 2022, the distribution of spatiotemporal clusters of LSD, disease risk factors, and high-risk areas in Asian continent were investigated. To ensure the integrity of the scope of the study, we discarded political considerations and national boundaries and included the entire Russian Federation in the study of the Asian continent.

In this study, nine spatiotemporal clusters were discovered in the Asian continent, which were located in Turkey and the Middle East, the Russian Caucasus, the Russian-Kazakhstan border, Mongolia, China, India, and Southeast Asia. Before 2019, the spatiotemporal clusters of LSD outbreaks were mainly concentrated in the Russian Caucasus and the Middle East [33]. After 2019, the epidemic gradually spread to central Asia (mainly China, Mongolia, and India) and Southeast Asia, which was reflected in the spatiotemporal analysis of LSD spread. However, these spatiotemporal clusters also corresponded to the risk areas of the disease. Some high-risk areas were consistent with those reported in previous studies [34].

LSD outbreaks show a seasonality pattern, and this pattern is caused by the mechanical transmission of arthropod vectors; hence, bioclimatic variables were selected as predictors in the maximum entropy model [7, 35]. Isothermality and maximum temperature of the warmest month were the critical bioclimatic variables that affected LSD outbreaks, with variable contribution rates of 9.2% and 8.0%, respectively. The corresponding curve showed that the risk probability of LSD outbreaks increased with the increase in isothermality. The risk probability was the highest at 85% isothermality. Isothermality is the ratio of the average daily temperature difference and the average annual temperature difference. The larger the ratio, the less obvious is the change in annual temperature, which enables most arthropods to survive throughout the year [36]. It was also observed that the risk of LSD outbreaks was the maximum when the maximum temperature of the warmest month reached 35°C. Temperature greatly affects the living habits of cattle [37]. In tropical and subtropical regions, cows do not need to be protected from cold and to be kept warm. They can stay active outdoors throughout the year. Some areas even adopt the approach of free grazing for cows, which greatly increases the probability of contact with bloodsucking insects. After bloodsucking insects bite the diseased cattle, they carry the virus and spread it farther because of their wide range of activities. The warm climate is also more favorable to the growth and survival of arthropods. In temperate regions, the winter temperature is low, and the number of arthropods is significantly smaller and mainly concentrated indoors. Moreover, cattle are also maintained indoors to keep them warm and protect them from cold. Consequently, in temperate regions, the small range of activities of the host and the vector is not favorable for disease outbreak and spread.

Among the land type variables, the land cover showed a high contribution rate (10.9%). Land cover is a categorical variable that includes the following land types: closed to open (>15%) grassland or woody vegetation on regularly flooded or waterlogged soil; areas with fresh, brackish, or saline water which are most suitable for disease occurrence. These areas, with grassland and abundant water resources, are suitable for the survival of blood-sucking insects and grazing, and the density of blood-sucking insects is large. Consequently, in such situations, the coexistence of vectors such as blood-sucking insects and cattle in pastoral areas greatly increases the risk of disease occurrence and transmission [38, 39]. The probability of using a common drinking water source for cattle has also increased. Furthermore, previous studies have shown that the sharing of drinking water sources by cattle in gathering places is one of the important reasons for LSD spread among the entire population [40].

Among the population density variables, the variable contribution rate of cattle density was 53.8%, which was much higher than that of buffalo density (8.9%). An interesting finding based on the response curve of the variable was that the probability of disease occurrence decreases as the cattle density increased, and the probability of disease occurrence tends to be stable with the continuous increasing of cattle density. Previous studies have shown that LSD hardly spread through direct contact between cattle [41, 42]. This phenomenon is well explained by the changing characteristics of the response curve of the variable.

In the present study, we developed a maximum entropy niche model to predict and map the high-risk areas of the LSD outbreaks. The results showed that the high-risk areas were approximately matched with the spatiotemporal clusters of the LSD outbreaks. Although the data used in our model do not include the latest incidence data from India, the results show that the southern part of India is a high-risk area for LSD outbreaks, which confirms the accuracy of our model's prediction. The model also shows that the northeast region of China and some countries in West Asia have a certain degree of risk of LSD occurrence. Although, there has been no outbreak of LSD in these areas thus far, it is essential to mitigate this threat with adequate precautions and effective treatment measures.

## 5. Conclusions

The present study used data on the outbreak of LSD in the Asian continent from January 2012 to September 2022, combined with bioclimatic factors, land type, and population density, to predict the high-risk areas and risk factors for LSD outbreaks in this region. Isothermality, maximum temperature of the warmest month, cattle density, buffalo density, and land cover showed the highest variable contribution rates. The findings of the present study showed that the predicted high-risk areas approximately matched the spatiotemporal clusters of LSD. India is a high-risk area for LSD outbreaks, which is consistent with the outbreak of LSD reported in Indian media in September 2022; this confirmed that our model appropriately predicted the high-risk areas of LSD outbreaks, thus helping to formulate more targeted strategies to manage the outbreak and spread of LSD in Asia. Northeastern China and some countries in West Asia are also at a certain level of risk of LSD outbreaks and therefore require constant vigilance.

## Figures and Tables

**Table 1:** Details of the variables.

Variable code	Variable name	Source
bio_1	Annual mean temperature (°C)	WorldClim version 2.1
bio_2	Mean diurnal range (°C)	WorldClim version 2.1
bio_3	Isothermality (BIO2/BIO7) (×100)	WorldClim version 2.1
bio_4	Temperature seasonality (standard deviation × 100)	WorldClim version 2.1
bio_5	Max temperature of warmest month (°C)	WorldClim version 2.1
bio_6	Min temperature of coldest month (°C)	WorldClim version 2.1
bio_7	Temperature annual range (BIO5-BIO6) (°C)	WorldClim version 2.1
bio_8	Mean temperature of wettest quarter (°C)	WorldClim version 2.1
bio_9	Mean temperature of driest quarter (°C)	WorldClim version 2.1
bio_10	Mean temperature of warmest quarter (°C)	WorldClim version 2.1
bio_11	Mean temperature of coldest quarter (°C)	WorldClim version 2.1
bio_12	Annual precipitation (mm)	WorldClim version 2.1
bio_13	Precipitation of wettest month (mm)	WorldClim version 2.1
bio_14	Mean precipitation of driest month (mm)	WorldClim version 2.1
bio_15	Precipitation seasonality (CV)	WorldClim version 2.1
bio_16	Precipitation of wettest quarter (mm)	WorldClim version 2.1
bio_17	Precipitation of driest quarter (mm)	WorldClim version 2.1
bio_18	Mean precipitation of warmest quarter (mm)	WorldClim version 2.1
bio_19	Mean precipitation of coldest quarter (mm)	WorldClim version 2.1
Srad	Solar radiation (kJ/day)	WorldClim version 2.1
LC	Land cover	ESACCI Land cover website
Wind	Wind speed (m/s)	WorldClim version 2.1
Cattle	Cattle density	Livestock geo-wiki
Buffalo	Buffalo density	Livestock geo-wiki
NDVI	Normalized difference vegetation index	National Tibetan plateau third pole environment data center

**Table 2:** Specific classification of land cover types.

Value	Label
11	Postflooding or irrigated croplands (or aquatic)
14	Rainfed croplands
20	Mosaic cropland (50–70%)/vegetation (grassland/shrubland/forest) (20–50%)
30	Mosaic vegetation (grassland/shrubland/forest) (50–70%)/cropland (20–50%)
40	Closed to open (>15%) broad-leaved evergreen or semideciduous forest (>5 m)
50	Closed (>40%) broad-leaved deciduous forest (>5 m)
60	Open (15–40%) broad-leaved deciduous forest/woodland (>5 m)
70	Closed (>40%) needle-leaved evergreen forest (>5 m)
90	Open (15–40%) needle-leaved deciduous or evergreen forest (>5 m)
100	Closed to open (>15%) mixed broad-leaved and needle-leaved forest (>5m)
110	Mosaic forest or shrubland (50–70%)/grassland (20–50%)
120	Mosaic grassland (50–70%)/forest or shrubland (20–50%)
130	Closed to open (>15%) (broad-leaved or needle-leaved, evergreen or deciduous) shrubland (<5 m)
140	Closed to open (>15%) herbaceous vegetation (grassland, savannas or lichens/mosses)
150	Sparse (<15%) vegetation
160	Closed to open (>15%) broad-leaved forest regularly flooded (semipermanently or temporarily)-fresh or brackish water
170	Closed (>40%) broad-leaved forest or shrubland permanently flooded-saline or brackish water
180	Closed to open (>15%) grassland or woody vegetation on regularly flooded or waterlogged soil-fresh, brackish or saline water
190	Artificial surfaces and associated areas (Urban areas >50%)
200	Bare areas
210	Water bodies
220	Permanent snow and ice
230	No data (burnt areas, clouds…)

**Table 3:** Details of the variables that built the model.

Variable cord	Variable name	Source
Cattle	Cattle density	Livestock geo-wiki
LC	Land cover	ESACCI land cover website
Bio_3	Isothermality (BIO2/BIO7) (×100)	WorldClim version 2.1
Buffalo	Buffalo density	Livestock geo-wiki
Bio_5	Max temperature of warmest month	WorldClim version 2.1
ndvi	Normalized difference vegetation index	Resource and environment science and data center
Bio_18	Mean precipitation of warmest quarter	WorldClim version 2.1
Srad	Solar radiation	WorldClim version 2.1
Bio_15	Precipitation seasonality	WorldClim version 2.1
Bio_8	Mean temperature of wettest quarter	WorldClim version 2.1
Wind	Wind speed	WorldClim version 2.1
Bio_19	Mean precipitation of coldest quarter	WorldClim version 2.1

**Table 4:** Spatiotemporal cluster analysis of lumpy skin disease from 2012 to 2022 in the Asian continent.

Cluster	Center coordinates	Radius (km)	Time frame	Observed notifications	Expected notifications	*P* value
1	34.33824N, 46.44064E	1049.39	2014 to 2016	23619	1824.055717	<0.01
2	53.2186N, 40.6548E	1050.477	2016 to 2018	5282	288.4167044	<0.01
3	54.98289N, 72.12187E	592.5404	2018 to 2019	271	0.607214257	<0.01
4	27.09386N, 116.250072E	387.5792	2019 to 2020	216	6.939240719	<0.01
5	21.63652N, 86.857682E	907.7289	2021 to 2022	2154	69.81004667	<0.01
6	51.03526N, 107.068842E	1006.532	2021 to 2022	3856	3439.950399	<0.01
7	29.38111N, 71.64023E	978.472	2021 to 2022	45579	40661.17719	<0.01
8	14.70208N, 99.31154E	525.0999	2021 to 2022	244345	217980.9855	<0.01
9	1.80031N, 103.700998	1044.873	2021 to 2022	2717	2423.844718	<0.01

**Table 5:** Contribution rate of the 12 variables in the LSD MaxEnt model.

Variable cord	Variable name	Variable contribution (%)
Cattle	Cattle density	53.8
LC	Land cover	10.9
Bio_3	Isothermality (BIO2/BIO7) (×100)	9.2
Buffalo	Buffalo density	8.9
Bio_5	Max temperature of warmest month	8
ndvi	Normalized difference vegetation index	4.1
Bio_18	Mean precipitation of warmest quarter	1.8
Srad	Solar radiation	1.5
Bio_15	Precipitation seasonality	0.8
Bio_8	Mean temperature of wettest quarter	0.5
Wind	Wind speed	0.3
Bio_19	Mean precipitation of coldest quarter	0.3

## Data Availability

The datasets generated and/or analyzed during the study are available from the corresponding author on reasonable request.
